# DNA microarray-based detection of *Coxiella burnetii,* the causative agent of Q fever

**DOI:** 10.1186/1751-0147-56-27

**Published:** 2014-05-08

**Authors:** Gernot Schmoock, Ralf Ehricht, Lisa D Sprague

**Affiliations:** 1 Friedrich-Loeffler-Institut, Institut für Bakterielle Infektionen und Zoonosen, Naumburger Str. 96a, Jena 07743, Germany; 2 Alere, Löbstedter Str. 103, Jena 07749, Germany

**Keywords:** *Coxiella burnetii*, Q fever, Oligonucleotide microarray, Hybridisation, PCR, Zoonosis

## Abstract

**Background:**

An easy-to-handle microarray assay based on the cost-effective ArrayTube™ platform has been designed for the rapid and unequivocal identification of *Coxiella burnetii,* the causative agent of Q fever. The gene targets include the chromosomally coded markers *icd*, *omp*/*com*1, and IS*1111* as well as the plasmid coded markers *cbbE* and *cbhE*.

**Results:**

A representative panel comprising 50 German *C. burnetii* isolates and 10 clinical samples was examined to validate the test. All tested isolates harboured plasmid QpH1 and were correctly identified, corresponding to 100% sensitivity. The assay’s limit of detection was 100 genome equivalents (GE) for *icd*, *omp*/*com*1, *cbbE* and *cbhE* and 10 GE for IS*1111*. Assay specificity was 100% as determined by analysing a panel of 37 non-*Coxiella* strains.

**Conclusions:**

The present array is a rational assembly of established and evaluated targets for the rapid and unequivocal detection of *C. burnetii*. This array could be applied to the screening of vaginal swabs from small ruminants; screening of environmental samples e.g. on farms or screening of human samples.

## Background

The causative agent of the zoonosis Q fever, *Coxiella burnetii* is listed by the United States Centers for Disease Control and Prevention (CDC) as a Category B pathogen and potential bioterrorism agent [1]. This small, Gram-negative, nonmotile, obligate intracellular bacterium is highly infectious and experimental data suggest that less than 10 organisms can cause infection. In animals, infection takes mostly a subclinical or inapparent course but abortions or birth of weak offspring, especially in small ruminants, can occur [2,3]. In humans, the clinical picture can range from asymptomatic to severe, usually presenting with fever, severe headache, myalgia and fatigue, frequently accompanied by atypical pneumonia and/or hepatitis. Chronic Q fever, i.e. persistence of infection exceeding a period of six months duration, may lead to endocarditis, which can be fatal. Additionally, chronic hepatitis, osteomyelitis, and septic arthritis are known sequelae [4].

Clinical diagnosis of *C. burnetii* infections in man and animal usually relies on serology, despite molecular methods such as PCR-based assays being more suitable in terms of speed and specificity, especially within the first couple of weeks after onset of disease [5-7]. However, although PCR assays are generally very fast and sensitive, their multiplexing capacity is limited. Moreover, due to their high specificity, they are incapable of detecting e.g. novel species or variants of a known species [8]. Microarrays on the other hand can be designed with a multitude of different probes either suitable for species identification by using highly specific probes, or for the detection of related or novel species by using probes lying within conserved regions [8]. A further benefit of the multiple targets on an array is that they can partly mitigate the weakness of diagnostic PCR assays when the PCR primer target contains point mutations. These mutations can be present in variants within a species and can lead to false negative results.

In this study, we describe a microarray-based method adapted to the ArrayTube™ (AT) platform, using three chromosomal (*icd*, *omp*/*com*1, and IS*1111*) and two plasmid (*cbbE*, *cbhE*) coded targets for the detection of *C. burnetii*. This platform has repeatedly been shown to be suitable for detecting other biological agents such as *Burkholderia mallei/pseudomallei*, *Brucella* spp., *Bacillus anthracis,* and *Chlamydia* spp. [9-12], for bacterial species differentiation and genotyping [13] and for automation-based applications.

## Methods

### Cell culture and bacterial isolates

The heat inactivated preparations of *C. burnetii* isolates and clinical samples used in this study were obtained from the National Reference Laboratory of Q Fever at the Federal Research Institute for Animal Health (Friedrich-Loeffler-Institut (FLI), Jena, Germany) [14] (Table 1).

**Table 1:** **Panel of tested *Coxiella *** **isolates and clinical samples isolated in Germany**

**Year**	**Identifier**	**Host**	**Sample**	**Year**	**Identifier**	**Host**	**Sample**
1997	DP677	fallow deer	isolate	2009	DP758	goat	isolate
1997	DP684	cattle	isolate	2009	DP792	sheep	isolate
1997	DP822	goat	isolate	2009	DP795	sheep	isolate
1998	DP682	goat	isolate	2009	DP798	sheep	isolate
1998	DP685	tick	isolate	2009	DP801	sheep	isolate
1998	DP751	sheep	isolate	2009	DP804	sheep	isolate
1998	DP752	sheep	isolate	2009	DP807	sheep	isolate
1998	DP759	tick	isolate	2009	DP810	sheep	isolate
1999	DP683	sheep	isolate	2009	DP813	sheep	isolate
1999	DP730	cattle	isolate	2010	12Q1649	cattle	isolate
2001	DP678	sheep	isolate	2011	12Q1650	sheep	isolate
2001	DP748	cattle	isolate	2011	12Q1651	sheep	isolate
2001	DP753	sheep	isolate	2011	12Q1652	sheep	isolate
2002	DP734	cattle	isolate	2011	12Q1653	sheep	isolate
2002	DP791	sheep	isolate	2011	12Q1654	sheep	isolate
2003	DP676	human	isolate	2011	12Q1655	sheep	isolate
2003	DP679	cattle	isolate	2011	12Q1656	sheep	isolate
2003	DP680	sheep	isolate	2012	12Q1657	cattle	isolate
2003	DP749	cattle	isolate	2012	12Q1658	cattle	isolate
2003	DP754	sheep	isolate				
2004	DP681	goat	isolate				
2004	DP731	sheep	isolate	2008	15/24	sheep	vaginal swab
2004	DP732	sheep	isolate	2009	34/19	sheep	vaginal swab
2004	DP733	sheep	isolate	2009	34/23	sheep	vaginal swab
2004	DP735	cattle	isolate	2009	34/25	sheep	vaginal swab
2005	DP698	sheep	isolate	2009	34/51	sheep	vaginal swab
2007	DP819	cattle	isolate	2009	34/53	sheep	vaginal swab
2008	DP750	cattle	isolate	2009	34/54	sheep	vaginal swab
2008	DP756	goat	isolate	2011	11Q3592	sheep	vaginal swab
2008	DP790	goat	isolate	2012	12Q0778	sheep	vaginal swab
2008	DP816	sheep	isolate	2013	13Q2139	sheep	vaginal swab

All non-*Coxiella* bacteria used in this study were obtained from the German Collection of Microorganisms and Cultures (DSMZ, Braunschweig, Germany), and from the strain collection of the Institute of Bacterial Infections and Zoonoses at the Federal Research Institute for Animal Health (FLI, Jena, Germany). Bacteria were grown on standard media under conditions recommended by the respective bacterial strain collections. DNA from *Bartonella* spp, *Chlamydia* spp, *Francisella* spp, *Salmonella typhimurium*, *Haemophilus influenzae*, and *Legionella pneumophila* was obtained from the Institute for Medical Microbiology and Infection Control, Goethe University, Frankfurt/M., from the National Reference Laboratory of Psittacosis, from the National Reference Laboratory of Tularaemia and from the National Reference Laboratory of Salmonellosis at the FLI, Jena, from IDEXX in Ludwigsburg, and from the Institute for Medical Microbiology, Jena, respectively (Table 2).

**Table 2:** **Panel of non-*Coxiella *** **strains used for specificity testing**

**Species**	**Isolate**	**Species**	**Isolate**
*Actinobacillus pleuropneumoniae*	ATCC 27088	*Haemophilus influenzae*	ATCC 9006
*Bacillus cereus*	ATCC 10876	*Klebsiella pneumoniae*	
*B. megaterium*	ATCC 14581	subsp*. pneumoniae*	DSM 30104
*B. subtilis*	ATCC 6633	*Lactobacillus ruminis*	DSM 20403
*B. thuringiensis*	ATCC 10792	*Legionella pneumophila*	
*Bartonella henselae*	Marseille	subsp. *pneumophila*	DSM 7513
*B. quintana*	JK-31	*Mannheimia haemolytica*	ATCC 33396
*Bordetella bronchiseptica*	ATCC 19395	*Oligella urethralis*	DSM 7531
*Brucella abortus*	ATCC 23448	*Pasteurella multocida*	DSM 5281
*B. melitensis*	ATCC 23456	*Proteus mirabilis*	DSM 4479
*B. suis*	ATCC 23444	*Pseudomonas aeruginosa*	ATCC 9027
*Burkholderia pseudomallei*	ATCC 23343	*P. alcaligenes*	ATCC 14909
*B. cepacia*	ATCC 25416	*P. fluorescens*	ATCC 13525
*B. mallei*	ATCC 23344	*P. polymyxa*	ATCC 842
*Chlamydia psittaci*	C1/97	*Rhodococcus equi*	DSM 20307
*C. abortus*	07 DC0059	*Salmonella typhimurium*	9098
*Escherichia coli*	DSM 30083	*Staphylococcus aureus*	DSM 6732
*Francisella tularensis* subsp*.*		*Streptococcus equinus*	DSM 20558
*tularensis*	SchuS4/ FSC 237	*S. parauberis*	DSM 6631
*F. tularensis* subsp. *holartica*	LVS/ FSC 155	*Yersinia enterocolitica*	
		subsp. *palearctica* (Y 11)	DSM 13030

ATCC: American Type Culture Collection; DSM: Deutsche Stammsammlung für Mikroorganismen, Germany; FSC: Francisella Isolate Collection, Sweden.

### DNA extraction and quantification

Genomic DNA from inactivated preparations of *C. burnetii* isolates and from non-*Coxiella* bacteria was isolated using the High Pure PCR Template Preparation Kit™ (Roche Diagnostics, Mannheim, Germany) according to the manufacturer’s instructions. Quality and purity of the DNA were determined using a Nanodrop ND-1000 spectrophotometer (PEQLAB Biotechnologie GmbH, Erlangen, Germany). DNA quantification was performed with a TaqMan based real-time PCR assay targeting the transposase element IS*1111* or the isocitrate dehydrogenase gene (*icd*) as described by Klee *et al*. [15] and Hildebrandt *et al*. [16]. DNA quantification for NineMile RSA493 was performed with the IS*1111* real-time PCR assay and genome equivalents (GE) were calculated with 20 IS*1111* copies per genome. Priscilla Q177 DNA was quantified with an *icd* real-time PCR assay and GE calculation was done with one *icd* copy per genome. Reaction conditions have been described previously [16] with the exception of the master mix (Maxima™ Probe qPCR Master Mix, Fermentas, St. Leon-Rot, Germany) and thermocycler (Mx3000P Thermocycler, Agilent Technologies, Santa Clara, CA, USA).

### Primer and probe design

Gene specific primers and probes were designed and optimised using the Array Design software package (Alere Technologies GmbH, Jena, Germany) and the published target sequences from the reference strains NineMile RSA493 (GenBank: AE016828), Henzerling RSA331 and Priscilla Q177. After design, all primers and probes were blasted against the complete GenBank™ database (http://www.ncbi.nlm.nih.gov/BLAST/).

### Microarray layout and fabrication

**Figure 1:**
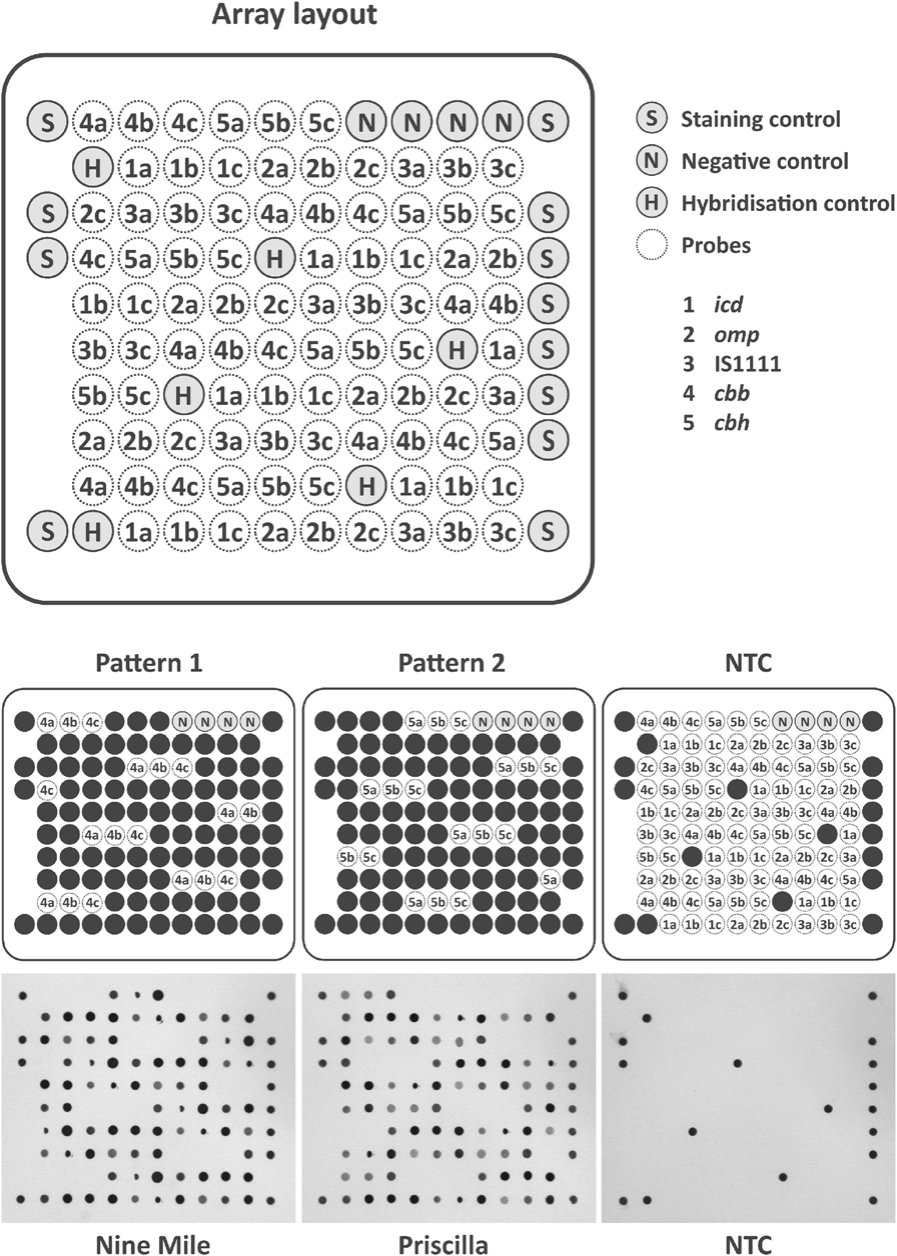
**Array layout, probe location, hybridisation patterns and hybridisation images for *Coxiella burnetii *** **strains NineMile RSA493, Priscilla Q177 and the non-template control (HPLC grade H**_**2**_**O).**

Oligonucleotides were purchased as 3'-amino-modified oligonucleotides from metabion (Martinsried, Germany) and diluted in Spotting Buffer 1 (Quantifoil Micro Tools, Jena, Germany) to a final concentration of 10 μM. Arrays were spotted with six-fold redundancy on surface-coated glass using Alere Technologies spotting machines and assembled into array tubes as described previously [17,18]. Internal staining controls (3’-amino and 5’-biotin-modified oligonucleotides) were included for staining control and orientation, as well as negative controls (0.1 M Sodium Pyrophosphate (NaPP) Standard pH 9). Probe sequences and array layout are shown in Table 3, and Figure 1, respectively.

**Table 3:** Sequences of oligonucleotide probes printed onto the microarray

**Probe**	**Target gene**	**Sequence (5’ → 3’)**	**Tm (°C)**	**GC (%)**	**Length (nt)**
1a	*icd*	CCTGACCGACCCATTATTCCCTTTATCGAAGGAG	66,1	50,0	34
1b		CCTGACCGACCCATTATTCCCTTTATCGAAGG	65,1	50,0	32
1c		CCCATTATTCCCTTTATCGAAGGAGATGGGATTGG	64,9	46,0	35
2a	*omp*/*com1*	CGTGGCGATAGCCGCCCCCTCTCAAT	69,3	65,0	26
2b		CTCCTCAACAAGTCAAAGACATACAAAGCATCG	63,3	42,0	33
2c		CAAAGCATCGTCCACCATTATTTAGTCAACC	62,0	42,0	31
3a	IS1111	GTAAAGTGATCTACACGAGACGGGTTAAGCG	64,0	48,0	31
3b		GCTCAGTATGTATCCACCGTAGCCAGTCTTAAG	64,6	48,0	33
3c		CTGCGTGGTGATGGAAGCGTGTGGAG	66,9	62,0	26
4a	*cbbE*	CTAAGAAACTGCTTAAGAGAGGGCAGGGAACG	65,3	50,0	32
4b		GGGCAGGGAACGATAGTGTGTTGCGGTATTTAC	67,3	52,0	33
4c		CTTGAAAAACGTAGCGGAAAAGGGACAGTACG	65,0	47,0	32
5a	*cbhE*	GCTTATTTCGCCCTCGCTGACGGAAGAGGATC	68,6	56,0	32
5b		CGGAAGAGGATCTTTTAGGTCGAATTAGACAGAGATAC	64,0	42,0	38
5c		GCTGGGGGCCAAGAAAAGTTTGCAGACCAG	68,5	57,0	30
H	HybCtr	GACTTACCAACACATCAAAGTTCCCAGC	61,7	46,0	28

### PCR amplification of target genes and generation of hybridisation control DNA

The 20 μL PCR reaction mixture contained 1 x 5 PRIME HotMasterMix (VWR International GmbH, Darmstadt, Germany), 0.3 μM forward primer, and 0.3 μM 5’-end biotinylated reverse primer (*icd*: AT_ICD-F/R, *omp/com*1: AT_Omp-F/R, IS*1111*: AT_IS-F/R, *cbb*E: AT_CbbE-F/R, *cbb*E: AT_CbhE-F/R, Table 4). Amplifications were performed using a Mastercycler ep® (Eppendorf, Germany). The reaction was started with a 1 min denaturation step at 96°C, followed by 35 cycles with 15 s of denaturation at 96°C, 20 s for annealing at 55°C, and 30 s for extension at 70°C. After a final 5 min extension step at 70°C the reaction was stopped and the PCR products submitted to electrophoresis. Hybridisation control DNA was generated from 10^5^ GE of the *C. burnetii* type strain (NineMile RSA493) using the PCR conditions above and the primer pair HybCtr-F (biotinylated) and HybCtr.

**Table 4:** Primers used for amplification of target genes and hybridisation control

**Name**	**Target gene**	**Sequence (5’ → 3’)**	**PCR product size (bp)**	**Tm (°C)**	**GC (%)**	**Length (nt)**
AT_ICD-F	*icd*	CGGAGTTAACCGGAGTATCCATC	178	57,6	52,0	23
AT_ICD-R		GCATCGACCACGTTTTTCATG		56,2	48,0	21
AT_Omp-F	*omp*/*com1*	GCACTATTTTTAGCCGGAACC	144	54,9	48,0	21
AT_Omp-R		TGCTTCTACTAAAACTTCTGGG		53,0	41,0	22
AT_IS-F	IS*1111*	CTGTGTGGAATTGATGAGTGG	142	54,2	48,0	21
AT_IS-R		ACGTCCGATACCAATGGTTC		55,0	50,0	20
AT_CbbE-F	*cbbE*	TAAGGGACATCCACTACCGG	147	55,5	55,0	20
AT_CbbE-R		CCCAAATTTAGATCGTCACATTG		53,6	39,0	23
AT_CbhE-F	*cbhE*	CGATGTCAACTCTAGAGAGC	147	52,5	50,0	20
AT_CbhE-R		GCAATCTGCTCGGCAATAAAG		55,6	48,0	21
HybCtr-F	ICD (HybCtr)	CGGAGTTAACCGGAGTATCCATC	95	57,6	52,0	23
HybCtr-R		AACTTCTAAAACGGCTTTATTAAC		51,5	29,0	24

### Hybridisation and data analysis

Hybridisation was carried out at 55°C for 60 min [10]. The hybridisation reaction was monitored using the ATR-01™ array tube reader (Alere Technologies) at 25°C for 10 min, recording one image at the end of incubation time. Signal intensity data with local background correction were obtained using the Iconoclust™ software, version 3.0 and Pionir/PARTISAN™ arrayLIMS software (Alere Technologies GmbH). The normalised intensity (NI) was calculated for each spot using the following equation: NI = 1-(*M*/BG), with *M* being the average intensity of the recognised spot and BG the intensity of its local background (Pionir/PARTISAN™ software). Values <0.15 were considered negative, values ≥0.15 positive.

### Determination of assay specificity and detection limit

In order to assess assay specificity, DNA from a panel of 37 type or in-house reference strains of different bacterial origin was submitted to the above described PCR procedure and subsequent hybridisation (Table 2). The detection limit was the smallest amount of *Coxiella* DNA detected by the test system for each individual target, and was determined in duplicate using tenfold serial dilutions containing 10^5^ to 10^0^ genome equivalents (GE) of NineMile RSA493 and Priscilla Q177, respectively.

## Results

### PCR amplification of target genes

Using the reference strains NineMile RSA493 (GenBank: AE016828), Henzerling, and Priscilla Q177, five separate PCR assays where established and optimised for each target (Table 3). All gene targets were successfully amplified and the obtained fragment sizes corresponded to the theoretical values (Table 4) calculated by *in silico* analysis of the respective target sequences (data not shown).

### Oligonucleotide probes and microarray layout

The selection of the microarray probe panel was based on five well characterised genetic markers of *C. burnetii*. The chromosomal target regions (*icd*, *omp*/*com*1, IS*1111*) were chosen due to their genetic stability. The plasmid coded target regions (*cbbE*, *cbhE*) were selected to identify the *Coxiella*-specific plasmids QpRS and QpH1 [19,20]. After initial BLAST analysis, all available sequences for the specific targets were used for sequence alignments. The final microarray layout consisted of 16 oligonucleotide probes of 26-38 bp in length with six-fold redundancy (Figure 1).

### Hybridisation results

Initially, hybridisation experiments were done with three reference strains (Nine Mile RSA493, Henzerling RSA331 and Priscilla Q177). Biotin-labelled amplification products of every individual target were pooled and applied to the array. The hybridisation patterns of all three reference strains complied with two patterns; these had been predicted from the *in silico* analysis of target region sequences derived from the GenBank™ sequence database: pattern 1 for RSA493 and RSA331, and pattern 2 for Q177, respectively (Figure 1).

In order to validate the new diagnostic assay, German *C. burnetii* isolates obtained from the German Q-fever network (http://www.fli.bund.de/de/startseite/institute/institut-fuer-bakterielle-infektionen-und-zoonosen/projekte/bmbf-verbundprojekt-q-fieber.html), and from the German National Reference Laboratory for Q fever were tested; a sample panel of 50 different isolates and 10 clinical samples (vaginal swabs) collected in Germany between 1997 and 2013 from sheep, goats, cattle, fallow deer, ticks, and humans were applied to the array. All analysed isolates revealed hybridisation signals corresponding to pattern 1.

### Determination of detection limits and assay specificity

The assay detection limit of every target was tested with RSA493 and Q177 in serial dilutions of DNA. Every marker was reliably detected down to DNA amounts of 100 genome equivalents (GE), whereby IS*1111* detection was more sensitive with a detection limit of 10 GE (data not shown). The assay specificity was evaluated with 37 type or in-house reference strains of various bacterial species (Table 2). No signals were observed in any of the hybridisation experiments with the test panel and internal hybridisation controls, thus confirming 100% assay specificity.

## Discussion

Diagnosis of Q fever in animal and man usually relies on serological methods, such as indirect immunofluorescence, complement fixation or enzyme-linked immunosorbent assays [5,21]. However, since *C. burnetii*-specific antibodies only begin to appear several weeks after infection, diagnosis is delayed. DNA based diagnostic assays are therefore more appropriate in terms of speed and specificity, and a plethora of PCR assays suitable for the detection of *C. burnetii* have been developed [15,22]. Although these assays are usually fast and sensitive and therefore more than adequate for most diagnostic applications, their capacity for multiplexing is limited [8]. This shortcoming has been resolved by using different types of DNA microarrays which have proved to be suitable for a broad range of applications in the microbial research and diagnostics of Q fever [7,23,24].

The microarray used in the present study was designed to target the chromosomally coded single copy genes isocitrate-dehydrogenase *icd*, the outer membrane protein-coding gene *omp*/*com*1 and the transpoase gene in the multi copy insertion element IS*1111* as well as the plasmid coded markers *cbbE* on QpRS and *cbhE* on QpH1. All these markers have been characterised extensively and their suitability as targets for detecting *C. burnetii* has been confirmed in numerous studies [19,25-29].

The analytical sensitivity of the assay used in the present study was 100 GE for the single copy gene targets *icd* and *omp*/*com*1 as well as for the plasmid coded targets *cbbE* and *cbhE* and 10 GE for the IS*1111*. Comparable values have been reported by Janse *et al*. [7], for *icd* and IS*1111* using suspension microarrays and for IS*1111* in a multiplex PCR targeting *icd*, *omp*/*com*1 and IS*1111* by de Bruin *et al*., [29]. The specificity of the array was 100% with the tested non-*Coxiella* strains, including the phylogenetic neighbours *Legionella* spp. and *Francisella* spp. as well as with *Bartonella* spp. which have been reported to cross react with *Coxiella* in serological assays [30]. We did not observe any cross reactions between probes or primers and sample DNA as reported by Janse *et al*. [7], which could be due to the different oligonucleotide design and different array chemistry.

*C. burnetii* can harbour four different plasmids of different size and composition, namely QpH1, QpRS, QpDG, QpDV and the chromosomally integrated plasmid-like sequences IPS [25,31,32]. Their role in the biology of *C. burnetii* is still not clear, but some plasmid genes appear to be essential for conserved functions [33]. Moreover, a potential influence of the different *C. burnetii* plasmids on animal and human disease has been discussed [34]. Early studies suggested that the plasmids QpH1 and QpRS are markers for acute or chronic disease, respectively [32], but this assumption could not be verified in a later study [35]. A small, recent study testing the hypothesis that obstetric complications in *C. burnetii* infections were associated with a particular genotype and the presence of QpDV, found that this plasmid type was detected more frequently in isolates associated with abortions [36]. All samples tested in the present study originated from Germany and harboured the plasmid QpH1. This plasmid type seems to be the predominant type in the isolates circulating in Germany and allegedly the Netherlands [14,36]. Since the present array only includes probes for the plasmids QpH1 and QpRS, which currently appear to be the most frequently occurring plasmid types in *C. burnetii*[14,35], the existing array could be improved by implementing additional probes for the other known plasmids of *C. burnetii*.

Several arrays have been developed for the detection of *C. burnetii*[7,23,36,37]. These arrays range from easy-to-handle, cheap but inflexible in terms of further probe implementation, to highly sophisticated, expensive-on-purchase but flexible regarding assay design. The ArrayTube™ platform is an easy-to-handle, middle price range (approx. 15 Euro/Array and PCR), molecular test for high-throughput and parallel analysis of samples. Array designs can be readily expanded by adding further gene targets. Moreover, the modular composition of the platform allows the rapid assembly of custom-made assays, targeting different biological agents, e. g. as part of a Bioweapon-agent-array detecting *C. burnetii*, *Brucella* spp., *B. mallei*/*pseudomallei, B. anthracis* and *Chlamydia* spp*.*[9-12].

## Conclusions

The present array is a rational assembly of established and evaluated targets for the rapid and unequivocal detection of *C. burnetii*. This array could be applied to the screening of vaginal swabs from small ruminants, screening of environmental samples e.g. on farms, for screening patients with infective endocarditis [38], or of blood donors in regions of high endemicity for Q fever, e.g. the German-Dutch border area, or in a diagnostic assay screening for atypical pneumonias [23].

## Abbreviations

AT: Array tube; GE: genome equivalent; IS1111: Insertion element 1111; icd: Isocitrate dehydrogenase; omp/com1: Outer membrane protein; NTC: Non-template control; PCR: Polymerase chain reaction; HPLC: High performance liquid chromatography.

## Competing interests

GS and LDS have no financial or other competing interests. RE is an employee of Clondiag/Alere, the producer of the ArrayTube™ platform.

## Authors’ contributions

RE and GS conceived and designed the experiments. GS analysed the data. LDS drafted and wrote the manuscript. All authors have read and approved the final version of the manuscript.
